# Chronic Fine and Coarse Particulate Exposure, Mortality, and Coronary Heart Disease in the Nurses’ Health Study

**DOI:** 10.1289/ehp.0900572

**Published:** 2009-06-15

**Authors:** Robin C. Puett, Jaime E. Hart, Jeff D Yanosky, Christopher Paciorek, Joel Schwartz, Helen Suh, Frank E Speizer, Francine Laden

**Affiliations:** 1 South Carolina Cancer Prevention and Control Program and; 2 Departments of Epidemiology and Biostatistics and Environmental Health Sciences, Arnold School of Public Health, University of South Carolina, Columbia, South Carolina, USA; 3 Exposure, Epidemiology, and Risk Program, Department of Environmental Health, Harvard School of Public Health, Boston, Massachusetts, USA; 4 Channing Laboratory, Department of Medicine, Brigham and Women’s Hospital and Harvard Medical School, Boston, Massachusetts, USA; 5 Department of Biostatistics, Harvard School of Public Health, Boston, Massachusetts, USA; 6 Department of Statistics, University of California at Berkeley, Berkeley, California, USA; 7 Department of Epidemiology, Harvard School of Public Health, Boston, Massachusetts, USA

**Keywords:** air pollution, cardiovascular disease, mortality, particulate matter

## Abstract

**Background:**

The relationship of fine particulate matter < 2.5 μm in diameter (PM_2.5_) air pollution with mortality and cardiovascular disease is well established, with more recent long-term studies reporting larger effect sizes than earlier long-term studies. Some studies have suggested the coarse fraction, particles between 2.5 and 10 μm (PM_10–2.5_), may also be important. With respect to mortality and cardiovascular events, questions remain regarding the relative strength of effect sizes for chronic exposure to fine and coarse particles.

**Objectives:**

We examined the relationship of chronic PM_2.5_ and PM_10–2.5_ exposures with all-cause mortality and fatal and nonfatal incident coronary heart disease (CHD), adjusting for time-varying covariates.

**Methods:**

The current study included women from the Nurses’ Health Study living in metropolitan areas of the northeastern and midwestern United States. Follow-up was from 1992 to 2002. We used geographic information systems–based spatial smoothing models to estimate monthly exposures at each participant’s residence.

**Results:**

We found increased risk of all-cause mortality [hazard ratio (HR), 1.26; 95% confidence interval (CI), 1.02–1.54] and fatal CHD (HR = 2.02; 95% CI, 1.07–3.78) associated with each 10-μg/m^3^ increase in annual PM_2.5_ exposure. The association between fatal CHD and PM_10–2.5_ was weaker.

**Conclusions:**

Our findings contribute to growing evidence that chronic PM_2.5_ exposure is associated with risk of all-cause and cardiovascular mortality.

A substantial body of literature has shown associations of particulate air pollution with mortality and specifically cardiovascular disease. Recent studies have focused on the fine fraction < 2.5 μm in diameter (PM_2.5_) (Eftim et al. 2008; Laden et al. 2006; Miller et al. 2007). However, some studies have suggested that the coarse fraction, particles between 10 and 2.5 μm (PM_10–2.5_), may be important as well (Brunekreef and Forsberg 2005; Host et al. 2008; Lipsett et al. 2006). A recent study in the Women’s Health Initiative (WHI) reported a 24% increase in the risk of a cardiovascular event [hazard ratio (HR) = 1.24; 95% confidence interval (CI), 1.09–1.41] and a 76% increase in the risk of death from cardiovascular disease (HR = 1.76; 95% CI, 1.25–2.47) for each 10-μg/m^3^ change in PM_2.5_ levels measured in 2000 (Miller et al. 2007). The magnitude of these estimates is higher than those reported in most long-term follow-up studies (Laden et al. 2006; Pope et al. 2002). Neither Pope et al. (2002) nor Miller and coauthors (2007) observed evidence of a positive association with PM_10–2.5_.

We previously observed a positive association of chronic PM_10_ (particles < 10 μm in aerodynamic diameter) exposures and all-cause mortality and fatal coronary heart disease (CHD) in the Nurses’ Health Study (NHS), a prospective cohort study of U.S. women (Puett et al. 2008). In the current study, we extend that study to look specifically at exposures to PM_2.5_ and PM_10–2.5_ in the same population. This longstanding cohort provides a unique opportunity with biennial updated assessment of covariates to examine these associations. With the geocoding of the nurses’ biennially updated residential addresses and the recent development of geographic information systems (GIS)–based spatial smoothing models, this study uses time- and space-resolved PM_2.5_ and PM_10–2.5_ exposures at the monthly level. This individual-specific exposure assessment approach has not been possible in many previous studies of chronic air pollutant effects.

## Methods

### Study population

The NHS began in 1976 with 121,700 female registered nurses who lived in 11 states (California, Texas, Florida, Massachusetts, Pennsylvania, Ohio, New York, New Jersey, Michigan, Connecticut, and Maryland), were born between 1921 and 1946, completed a baseline questionnaire, and provided informed consent. The Brigham and Women’s Hospital Institutional Review Board approved all aspects of this study. Participants have been mailed biennial questionnaires to their residential address to obtain information on risk factors and health outcomes since the study’s inception. Among nurses available for follow-up, about 6% did not respond to current questionnaires. For the current study we included participants who were living from 1992 until 2002 in metropolitan statistical areas (MSAs) of 13 contiguous states in the northeast and midwest United States (Maine, Vermont, New Hampshire, Ohio, Pennsylvania, Maryland, Michigan, Massachusetts, Connecticut, Rhode Island, New York, New Jersey, Delaware). We chose to limit the study population to women residing in MSAs (about 87% of participants in this geographic region) to allow for comparisons with results from previous studies that also have focused on metropolitan areas (Eftim et al. 2008; Pope et al. 1995, 2002) and because the distributions of air pollution monitors and nurses were more sparse outside MSAs. Women were excluded for any time period of follow-up during which they resided outside this geographic region.

Nonfatal myocardial infarctions (MIs) were assessed through biennial questionnaires and confirmed through medical record review by physicians blinded to study participants’ exposure status. Deaths were obtained through next of kin, postal authority reports, death certificates, or the National Death Index. Fatal CHD was confirmed by death certificate, hospital records, or autopsy. Additional details regarding the assessment and confirmation of nonfatal MI, first-incident nonfatal or fatal CHD, fatal CHD, and all-cause mortality for the current study are described elsewhere (Puett et al. 2008). Only cases indicated as definitely or probably confirmed were counted. Women reporting cancers prior to 1992 (other than nonmelanoma skin cancer) were excluded at the beginning of follow-up. Accidental deaths were excluded from the all-cause mortality analysis. Women with nonfatal MIs prior to baseline were excluded from fatal and nonfatal incident CHD cases for the current study.

### Exposure assessment

Separate spatio-temporal models for PM_10_ and PM_2.5_ were developed and validated, with coarse particle levels estimated by subtraction of predicted PM_2.5_ from predicted PM_10_ (Paciorek et al. 2009; Yanosky et al. 2008, 2009).

The PM_10_ model is a GIS-based spatial smoothing model that predicts monthly outdoor concentrations specific to each participant’s biennially updated residence. This generalized additive mixed model [GAMM, detailed elsewhere (Paciorek et al. 2009; Yanosky et al. 2008)] used monitoring data from sites in the U.S. Environmental Protection Agency’s Air Quality System (U.S. EPA 2007), the Interagency Monitoring of Protected Visual Environments (IMPROVE) network (National Aeronautics and Space Administration 2009), Clean Air Status and Trends Network (CASTNet, U.S. EPA 2009) data, and Harvard research studies to estimate monthly smooth spatial terms and penalized regression terms of GIS-based and meteorologic covariates. These covariates included population density; distance to nearest road by Census Feature Class Code A1-3; elevation; urban land use; point- and area-source PM_10_ emissions; wind speed; and precipitation.

We followed a similar process to predict monthly outdoor PM_2.5_ concentrations (Paciorek et al. 2009; Yanosky et al. 2009). Briefly, because of the lack of PM_2.5_ monitor data before 1999, we constructed separate models for 1988–1998 and 1999–2002. The post-1999 PM_2.5_ model was of similar form to the PM_10_ model, and with similar covariates but used point-source PM_2.5_ emissions. The pre-1999 model used a simpler spatiotemporal structure to estimate the PM_2.5_ to PM_10_ ratio seasonally and used estimated extinction coefficients and covariates described previously. We estimated PM_10–2.5_ exposures by subtracting the modeled PM_2.5_ estimates from the PM_10_ modeled estimates for each month at each location. The PM_10_ model and post-1999 and pre-1999 PM_2.5_ models were validated using cross-validation. This procedure involved dividing the monitoring locations into 10 sets, and fitting the model with each set held out. Then, we calculated the squared correlation between held-out observations and predictions from the model with each set removed. We used sets 1–9 to assess model performance, whereas we reserved set 10 to evaluate model over-fitting. Each of these models performed well using cross-validation, exhibiting little bias and high precision (Paciorek et al. 2009; Yanosky et al. 2008, 2009). In comparison, predicted PM_10–2.5_ levels exhibited little bias but were less precise (cross-validation results are detailed elsewhere) (Yanosky et al. 2009).

### Evaluation of confounders and effect modifiers

Data from the biennial questionnaires were used to assess potential confounding and effect modification by covariates, including hypertension (yes, no); physician-diagnosed diabetes (yes, no); hypercholesterolemia (yes, no); physical activity (< 3, 3 to < 9, 9 to < 18, 18 to < 27, or ≥ 27 metabolic equivalent (MET) hr per week); body mass index (BMI) (continuous); smoking status (never, former, or current); and smoking pack-years. Family history of MI (yes, no) was included based on answers to the 1976 and 1984 questionnaires. Census 2000 data were used to assign two group-level socioeconomic status variables, median household income and median household value at the census tract level. Confounding was assessed through adjustment for each of these covariates in individual and multivariate Cox models. Effect modification was evaluated through stratification and the use of interaction terms.

### Statistical analysis

Time-varying Cox proportional hazards models were used to assess the relationship of all-cause mortality and CHD outcomes with PM_2.5_ and PM_10–2.5_. These models were based on a monthly time scale and were used to estimate HRs and 95% CIs. Person-months of follow-up time were calculated from baseline (30 June 1992) until the end of follow-up (30 June 2002), censoring at death or loss to follow-up. Person-time spent living outside the selected region was excluded, as were nurses with cancers or outcomes of interest (e.g., nonfatal MI) reported prior to baseline. Incidence rates were estimated as the number of new cases divided by person-months of follow-up. We focused on the average exposure to PM_2.5_ and PM_10–2.5_ in the 12 months prior to the outcome of interest because a previous study has shown that to be the most relevant exposure (Schwartz et al. 2008). However, in separate models, we also considered other time windows of exposure, including average exposure in the 1, 3, 24, 36, and 48 months prior to the event. We assessed PM_2.5_ and PM_10–2.5_ in single- and two-pollutant models. All Cox models were stratified by age in months and adjusted for state of residence (indicator variables), year (linear term), and season (indicator variables). By including state variables, the model adjusts for large-scale spatial patterns in mortality that might be caused by factors other than pollution, thereby estimating the effect of pollution based on within-state variation (Dominici et al. 2006; Pope et al. 2002). All statistical analyses used SAS version 9.1 (SAS Institute Inc., Cary, NC, USA).

## Results

The study population consisted of 66,250 women who lived in MSAs in the northeastern and midwestern United States in 1992 (Table 1) (Puett et al. 2008). Their mean age was 62.4 years. During the follow-up period, most were never or former smokers and 42% had a BMI under 25. Mean (± SD) levels of PM_2.5_ and PM_10–2.5_ exposures in the previous 12 months were 13.9 ± 2.4 and 7.7 ± 2.6 μg/m^3^, respectively (further details on PM described elsewhere) (Paciorek et al. 2009; Yanosky et al. 2009). There were 3,785 deaths; 1,348 incident CHD events; 379 fatal CHDs; and 854 nonfatal MIs.

HRs and 95% CIs for all-cause mortality and other outcomes of interest for a 10-μg/m^3^ unit change in PM_2.5_ and PM_10–2.5_ averaged over the previous 12 months are presented in Table 2. In models adjusted for age, calendar time, and state of residence, PM_2.5_ was significantly associated with all-cause mortality (HR = 1.45; 95% CI, 1.19–1.78). Results also suggest PM_10–2.5_ may be associated with increased mortality risk (HR = 1.13; 95% CI, 0.98–1.30). The HRs for both size fractions of PM and incident CHD were also elevated. Risks associated with fatal CHD were larger for PM_2.5_ (HR = 2.29; 95% CI, 1.26–4.18) than for PM_10–2.5_ (HR = 1.28; 95% CI, 0.82–1.98) in crude models adjusted for age, calendar time, and state of residence. In sensitivity analyses using average annual PM_2.5_ exposure estimates from the nearest U.S. EPA AQS monitor in 2000, as opposed to PM_2.5_ estimates from the time-varying geospatial model, the risks of all-cause mortality (HR = 1.35; 95% CI, 1.08–1.69) and fatal CHD (HR = 1.47; 95% CI, 0.73–2.99) were attenuated but elevated.

Fully adjusted models included hypertension, family history of MI, hypercholesterolemia, BMI, physical activity, smoking, diabetes, median house value, and household income for census tract of residence, season, and state of residence and were stratified by age in months (Table 2). Confounders were not highly correlated. The HRs were attenuated compared to models with limited control for confounding. PM_2.5_ was associated with all-cause mortality (HR = 1.26; 95% CI, 1.02–1.54) and fatal CHD (HR = 2.02; 95% CI, 1.07–3.78). Effect estimates for PM_2.5_ and PM_10–2.5_ were generally stable in two pollutant models, although estimates with all-cause mortality and fatal CHD were strengthened for PM_2.5_ and attenuated for PM_10–2.5_. Overall, for nonfatal MI, the effect strengthened for PM_10–2.5_.

**Figure 1:**
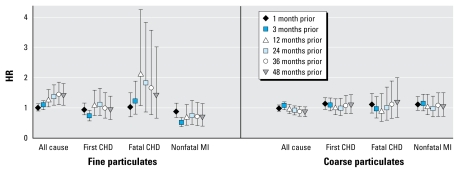
HRs and 95% CIs for the association between all-cause and cause-specific mortality and a 10-μg/m^3^ change in average PM_2.5_ and PM_10–2.5_ for six time periods of exposure. ***a***Fine and coarse PM levels modeled simultaneously, stratified by age in months, adjusted for state of residence, year and season, smoking status, family history of MI, BMI, hypercholesterolemia, diabetes, hypertension, median family income in census tract of residence, physical activity, and median house value in census tract of residence.

We assessed the sensitivity of our results to different time periods of exposure: 1, 3, 24, 36, and 48 months before the event. In single-pollutant, fully adjusted models of PM_2.5_ exposure, the associations with each outcome (except nonfatal MI) were stronger with times greater than 3 months and similar among time periods 12–48 months. In equivalent models for PM_10–2.5_, there were no apparent differences among exposure windows (data not shown). Results for different periods of exposure were similar for multipollutant fully adjusted models (Figure 1).

Table 3 shows relationships of PM_2.5_ exposures in the previous 12 months with all-cause mortality and fatal CHD adjusting for each potential confounder one at a time (after adjusting for state of residence, year, and season, and stratifying by age). For both fatal CHD and all-cause mortality, median house value for the census tract of residence elevated the risk, whereas physical activity attenuated the risk associated with PM_2.5_ exposures.

Although no interaction terms were statistically significant, stratified analyses for each covariate, adjusting for all other covariates, showed some differences in the association between all-cause mortality and chronic PM_2.5_ exposure (Table 4). Women with hypercholesterolemia or in the lowest category of physical activity were at higher risk. Risks were greatest for nonsmokers and least for current smokers. There was no evidence of effect modification for the relationship between all-cause mortality and PM_10–2.5_ (data not shown).

Women with a family history of MI were at significantly higher risk of fatal CHD associated with PM_2.5_ exposure (Table 4). Stratified analyses also suggested greater risks for women with hypertension, hypercholesterolemia, larger BMIs, and living in census tracts in the lowest quartile of median house value or the lowest two quartiles of median family income. Never smokers showed the highest risk and current smokers, the least. Similar stratified differences by BMI and smoking were evident for PM_10–2.5_ (data not shown).

## Discussion

In this study among women in the northeastern and midwestern region of the United States, we found each 10-μg/m^3^ elevation of PM_2.5_ exposure in the previous 12 months was associated with an increased risk of all-cause mortality (HR = 1.26; 95% CI, 1.02–1.54) and fatal CHD (HR = 2.02; 95% CI, 1.07–3.78) after controlling for known risk factors. Although we found evidence of a positive association between PM_10–2.5_ exposure and all-cause mortality, there was no association after adjustment for covariates. An association between fatal CHD and PM_10–2.5_ exposures was also evident but weaker in fully adjusted models. Finally, there was little evidence of an association between incident MI and PM. The relationship between PM_2.5_ and fatal CHD was modified by family history of MI, and nonsmokers were at greatest risk, suggesting the strong impact of smoking exposures conceals the effects of air pollution. However, CIs were wide. The attenuation of risk of all-cause mortality and fatal CHD by physical activity as well as the increased risk of all-cause mortality for women recording the least activity raises questions about the biological mechanism underlying these relationships. Few previous studies have examined the influence of physical activity.

These results are consistent with those observed in the growing body of literature on chronic air pollution and health effects. In the extended follow-up of the American Cancer Society (ACS) Study, a 10-μg/m^3^ change in PM_2.5_ was associated with an HR of 1.06 (95% CI, 1.02–1.11) for all-cause mortality (Pope et al. 2002). The equivalent HR for a 10-μg/m^3^ change in the updated Harvard Six Cities Study was 1.14 (95% CI, 1.06–1.22) (Laden et al. 2006). Recently, Eftim et al. (2008) replicated these analyses among Medicare patients residing in the same counties included in these two studies. Their results were more consistent with those observed in our cohort (ACS: HR for a 10-μg/m^3^ change = 1.11; 95% CI, 1.09–1.13; Six Cities: HR = 1.21; 95% CI, 1.15–1.27). Specific associations with CHD also have been consistently observed. The Harvard Six Cities (Laden et al. 2006) and ACS (Pope et al. 2002) studies observed associations of 1.28 (95% CI, 1.13–1.44) for cardiovascular mortality and 1.09 (95% CI, 1.03–1.16) for cardiopulmonary mortality, respectively. The recent WHI study reported overall risks of 2.21 (95% CI, 1.17–4.16) for cardiovascular mortality and 1.76 (95% CI, 1.25–2.47) for incident CHD (Miller et al. 2007). Two additional studies of men and women have found greater susceptibility among women for cardiovascular events associated with particulate matter exposures (Chen et al. 2005; Rosenlund et al. 2006).

Neither the original ACS study (Pope et al. 1995) nor the WHI study (Miller et al. 2007) observed an association between all-cause mortality and chronic exposure to PM_10–2.5_. However, the Harvard Six Cities study found an elevated relative risk associated with exposure to PM_15–2.5_ (HR = 1.19; 95% CI, 0.91–1.55) (U.S. EPA 1996). McDonnell et al. (2000) observed an HR of 1.05 (95% CI, 0.92–1.20) for an IQR increase (9.7 μg/m^3^) in PM_10–2.5_ among men living near an airport in a cohort of Seventh-day Adventists. In an acute exposure study of fine and coarse particles in Shanghai, China, Kan et al. (2007) found no significant effect of PM_10–2.5_ on mortality, but increases of total and cardiovascular mortality were reported. Other studies of acute exposure to coarse particulate matter have also suggested a relationship with cardiovascular outcomes (Host et al. 2008; Lipsett et al. 2006; Peng et al. 2008; Tolbert et al. 2000), although Peng et al. (2008) reported the association between daily CVD hospital admissions and coarse particulate matter was no longer statistically significant when adjusted for PM_2.5_. We found a stronger association between coarse particulate matter and fatal CHD than with all-cause mortality in fully adjusted single-pollutant models and with nonfatal MI in multipollutant models. In multipollutant models, the WHI also found an association of PM_2.5_ (HR = 1.53; 95% CI, 1.21–1.94) and PM_10–2.5_ (HR = 1.10; 95% CI, 0.97–1.23) with first cardiovascular event (Miller et al. 2007).

In general, our results are elevated in relation to other studies, with the exception of the WHI, another cohort study of women. This disparity could be due in part to the use of different air pollution exposure estimation methods. We modeled monthly exposures for each biennially updated residential address using GIS-based spatial smoothing models. For example, the ACS study used mean exposure in metropolitan areas measured during a few of the years of follow-up (Pope et al. 1995). A reanalysis restricting to subjects with monitors in their county of residence reported higher risks (Willis et al. 2003). Additionally, a study in Southern California using spatially estimated exposures reported stronger results (Jerrett et al. 2005). To the extent that our exposure modeling accounts for local variation that other studies do not, we might be capturing different sources of pollution resulting in different effect sizes. Further, our sensitivity analyses, using a less-precise exposure estimate, showed an attenuation of the effect size. Therefore, it appears that studies using spatially estimated exposure measures may produce higher risk estimates. This has important implications for risk assessment.

Because PM_10–2.5_ estimates were derived from PM_10_ and PM_2.5_ estimates, more uncertainty is associated. This may contribute to the lower effect estimates we observed for PM_10–2.5_, but Yanosky et al. (2009) show that long-term average PM_10–2.5_ was reasonably well estimated (cross-validation *R*^2^ = 0.63 and 0.65 for long-term post-1999 and pre-1999 PM_10–2.5_, respectively). To improve our exposure modeling, we focused on the northeastern and midwestern United States (63% of the total study population), an area with more uniformly distributed study population and monitors, and results may differ for other U.S. regions. In addition, we were unable to account for nurses who moved between the biennial questionnaires or for lengthy stays away from their residence in another geographic region. We estimated time- and space-resolved exposures with GIS-based smoothing models. Although smoothing reduces variability relative to measured concentrations, Gryparis et al. (2008) show this is a type of Berkson measurement error that should not cause substantial bias toward the null. Additionally, modeling allows us to assign exposures specific to biennially updated residential addresses for the entire period of follow-up. Thus, compared with using only monitor measurements, fewer participants are lost because of missing exposure data.

A strength of this study is that we have updated information on numerous covariates throughout the follow-up period. However, there does exist the possibility of residual confounding or confounding by unmeasured covariates and/or by additional pollutants.

Biological mechanisms for the relationship of particulate matter exposure with mortality and CVD have not yet been fully explained. Several mechanisms have been proposed, including changes in autonomic function, oxidative stress, and systemic inflammation leading to endothelial dysfunction, thrombosis, or atherosclerosis (Donaldson et al. 2001; Gurgueira et al. 2002; Pope et al. 2004; Pope and Dockery 2006; Utell et al. 2002). Although fine particles deposit deeper into the lung (Brunekreef and Forsberg 2005; Venkataraman and Kao 1999), some studies have shown coarse particles have a greater ability to stimulate inflammatory responses and macrophages cytokine production (Becker et al. 2002, 2005; Brunekreef and Forsberg 2005).

## Conclusions

Our findings contribute to growing evidence that annual exposure to particles is associated with increases in risk of all-cause and cardiovascular mortality. The extended follow-up of the Harvard Six Cities study (Laden et al. 2006) found mortality risks associated with exposure in the year of death were similar to those for the entire follow-up period. In another reanalysis, Schwartz et al. (2008) reported that the association was with exposure in the previous 2 years. A recent study of a Medicare cohort of MI survivors and PM_10_ exposure examined the effect of multiple lags of exposure on survival. Again, the effect of particles on mortality risk seemed to go back only a few years (Zanobetti and Schwartz 2007).

In summary, with chronic coarse and fine particulate exposures estimated on a finer spatial and temporal scale than in previous cohort studies (Dockery et al. 1993; Pope et al. 1995), we found PM_2.5_ was associated with increased risks of all-cause mortality and fatal CHD. Coarse particulate matter exposures were not associated with an increase in risk after control for confounders. In addition, our results suggest that health benefits may be evident within a few years of reductions in particle levels.

## Figures and Tables

**Table 1:** Characteristics of the study population in selected categories from the Nurses’ Health Study during the follow-up period 1992–2002.^a^

Characteristic	Statistic
No.	66,250
Age [years (mean ± SD)]	62.4 ± 7.6
BMI (%)	
< 25.0	42.3
25.0 to < 30.0	34.2
> 30.0	23.5
Smoking status (%)	
Never	43.6
Current	13.5
Former	42.9
Pack-years (mean ± SD)	24.8 ± 21.0
Hypercholesterolemia (%)	49.5
Diabetes (%)	7.4
Hypertension (%)	40.4
Family history of MI (%)	34.2
Physical activity (%)	
< 3 MET hr/week	22.1
3 to < 9 MET hr/week	23.8
9 to < 18 MET hr/week	21.0
18 to < 27 MET hr/week	12.8
≥27 MET hr/week	20.3
Predicted PM_2.5_ μg/m^3^	
Mean ± SD	13.9 ± 2.4
Minimum**–**maximum	5.8**–**27.6
IQR	12.0**–**15.6
Predicted PM_10–2.5_ μg/m^3^	
Mean ± SD	7.7 ± 2.6
Minimum **–** maximum	< 0.01**–**26.9
IQR	5.9**–**9.2
Median family income in thousands (mean ± SD)^b^	67.0 ± 24.1
Median house value in ten thousands (mean ± SD)^b^	16.6 ± 10.4

a Percentages based on complete information for participants.

b Estimated for census tract of residence using data from the U.S. Census Bureau (2000).

c Data from Puett et al. (2008).

**Table 2:** HRs (95% CIs) for outcomes associated with a 10-μg/m^3^ change in average predicted PM_2.5_ and PM_10–2.5_ exposure in the previous 12 months.^a^

					Fully adjusted models^b^			
			Crude models^c^		Single-pollutant models		Multipollutant models^d^	
	Cases	Person-months	PM_2.5_	PM_10–2.5_	PM_2.5_	PM_10–2.5_	PM_2.5_	PM_10–2.5_
All-cause mortality	3,785	606,752	1.45 (1.19–1.78)	1.13 (0.98–1.30)	1.26 (1.02–1.54)	1.03 (0.89–1.18)	1.29 (1.03–1.62)	0.96 (0.82–1.12)
First CHD	1,348	597,456	1.19 (0.85–1.65)	1.12 (0.89–1.42)	1.11 (0.79–1.55)	1.04 (0.82–1.32)	1.10 (0.76–1.60)	1.01 (0.78–1.31)
Fatal CHD^e^	379	597,456	2.29 (1.26–4.18)	1.28 (0.82–1.98)	2.02 (1.07–3.78)	1.14 (0.73–1.77)	2.13 (1.07–4.26)	0.91 (0.56–1.48)
Nonfatal MI	854	597,458	0.76 (0.50–1.15)	1.01 (0.75–1.36)	0.73 (0.48–1.12)	0.96 (0.71–1.30)	0.71 (0.44–1.13)	1.06 (0.77–1.47)

a The moving average calculated for the 12 months previous to each risk set.

b Models include age, state of residence, year, season, smoking status, family history of MI, BMI, hypercholesterolemia, diabetes, hypertension, median family income in census tract of residence, physical activity, and median house value in census tract of residence.

c Models stratified by age and adjusted for state of residence, year, and season.

d PM_2.5_ and PM_10–2.5_ modeled simultaneously.

e Excluding prior nonfatal MI.

**Table 3:** Adjusted HRs (95% CIs) for all-cause and CHD mortality associated with a 10-μg/m^3^ change in average PM_2.5_ in prior 12 months.

Model covariates	All-cause mortality	Fatal CHD	First CHD	Nonfatal MI
Time, residential state, season	1.45 (1.19–1.78)	2.29 (1.26–4.18)	1.19 (0.85–1.65)	0.76 (0.50–1.15)
Family history of MI	1.47 (1.20–1.79)	2.36 (1.29–4.31)	1.22 (0.87–1.69)	0.77 (0.51–1.18)
Hypercholesterolemia	1.46 (1.20–1.78)	2.34 (1.28–4.27)	1.21 (0.87–1.68)	0.77 (0.51–1.17)
Hypertension	1.44 (1.18–1.76)	2.29 (1.25–4.18)	1.18 (0.85–1.63)	0.75 (0.49–1.14)
BMI	1.44 (1.18–1.75)	2.25 (1.23–4.09)	1.21 (0.87–1.68)	0.79 (0.52–1.20)
Diabetes	1.44 (1.18–1.76)	2.26 (1.24–4.14)	1.17 (0.84–1.63)	0.75 (0.49–1.14)
Median household income	1.46 (1.20–1.78)	2.30 (1.26–4.17)	1.20 (0.87–1.67)	0.78 (0.51–1.17)
Median house value	1.56 (1.27–1.92)	2.60 (1.39–4.84)	1.28 (0.92–1.79)	0.81 (0.53–1.23)
Smoking	1.36 (1.11–1.66)	2.20 (1.20–4.02)	1.15 (0.83–1.61)	0.74 (0.49–1.13)
Physical activity	1.27 (1.04–1.55)	2.02 (1.11–3.70)	1.14 (0.82–1.59)	0.76 (0.50–1.16)
Full model^a^ excluding physical activity	1.36 (1.11–1.66)	2.15 (1.15–4.02)	1.14 (0.81–1.59)	0.74 (0.48–1.13)
Full model^a^	1.26 (1.02–1.54)	2.02 (1.07–3.78)	1.11 (0.79–1.55)	0.73 (0.48–1.12)

a All models adjusted for state of residence, year, and season and stratified by age (months); full model includes all listed covariates.

**Table 4:** All-cause and CHD mortality and first CHD associated with 10-μg/m^3^ change in average PM_2.5_ in prior 12 months, stratified by potential effect modifiers.

	All-cause mortality		Fatal CHD		First CHD	
Modifier	Cases	HR (95% CI)	Cases	HR (95% CI)	Cases	HR (95% CI)
Diabetes						
No	2,970	1.27 (1.01–1.61)	240	1.85 (0.83–4.11)	1,001	1.00 (0.68–1.49)
Yes	815	1.19 (0.77–1.85)	139	1.90 (0.67–5.43)	347	1.22 (0.63–2.37)

Family history of MI						
No	2,352	1.02 (0.79–1.32)	217	1.31 (0.57–3.00)	746	0.96 (0.61–1.51)
Yes	1,433	1.85 (1.32–2.60)	162	3.50 (1.33–9.24)*	602	1.28 (0.77–2.13)

Hypercholesterolemia						
No	1,798	1.04 (0.77–1.40)	154	1.46 (0.53–4.04)	494	1.23 (0.70–2.15)
Yes	1,987	1.53 (1.15–2.03)	225	2.38 (1.07–5.32)	854	1.03 (0.67–1.57)

Hypertension						
No	1,505	1.40 (1.01–1.94)	108	1.21 (0.37–4.01)	437	1.20 (0.66–2.17)
Yes	2,280	1.17 (0.90–1.53)	271	2.27 (1.07–4.79)	911	1.02 (0.68–1.54)

Smoking						
Never	1,091	1.45 (1.00–2.11)	126	4.55 (1.52–13.58)	445	1.18 (0.66–2.11)
Former	1,785	1.29 (0.95–1.75)	142	2.36 (0.83–6.73)	566	1.21 (0.71–2.05)
Current	791	1.04 (0.66–1.63)	98	0.71 (0.20–2.50)	314	1.04 (0.52–2.10)

Physical activity (MET hr)						
0–3	1,007	1.56 (1.04–2.34)	95	3.69 (1.03–13.23)	311	1.69 (0.83–3.44)
3–18	899	1.05 (0.69–1.60)	81	1.01 (0.25–4.01)	497	0.79 (0.45–1.40)
> 18	234	0.91 (0.40–2.09)	28	0.15 (0.01–1.58)	145	0.64 (0.23–1.79)

Median house value						
Quartile 1	866	1.16 (0.78–1.71)	98	3.63 (1.20–11.00)	345	0.94 (0.51–1.73)
Quartile 2	963	1.25 (0.83–1.88)	91	2.34 (0.75–7.32)	359	1.14 (0.59–2.20)
Quartile 3	1,007	1.07 (0.68–1.67)	102	1.41 (0.49–4.06)	353	1.04 (0.49–2.18)
Quartile 4	942	1.24 (0.76–2.02)	87	1.38 (0.41–4.66)	287	1.93 (0.81–4.59)

Median family income						
Quartile 1	888	1.04 (0.71–1.52)	97	2.33 (0.85–6.34)	335	0.68 (0.38–1.23)
Quartile 2	984	1.49 (1.00–2.21)	97	6.19 (1.98–19.40)	377	2.46 (1.31–4.62)
Quartile 3	1,023	1.03 (0.67–1.58)	102	0.61 (0.18–2.03)	354	0.72 (0.35–1.49)
Quartile 4	890	1.09 (0.66–1.81)	83	1.44 (0.30–6.91)	282	1.44 (0.59–3.51)

BMI						
< 30	2,817	1.30 (1.02–1.65)	248	1.09 (0.06–19.98)	927	0.85 (0.56–1.29)
> 30	827	1.76 (1.15–2.70)	115	3.02 (0.97–9.40)	389	1.97 (1.06–3.63)

The model was stratified by age in months, adjusted for state of residence, year and season, smoking status, family history of MI, hypercholesterolemia, diabetes, hypertension, BMI, median family income in census tract of residence, physical activity, and median house value in census tract of residence.

* Interaction significant at *p* < 0.05.
