# Correction: Chronic Fine and Coarse Particulate Exposure, Mortality, and Coronary Heart Disease in the Nurses’ Health Study

**DOI:** 10.1289/ehp.0900572-erratum

**Published:** 2010-02-01

Puett et al. found an error in their article “Chronic Fine and Coarse Particulate Exposure, Mortality, and Coronary Heart Disease in the Nurses’ Health Study” [Environ Health Perspect 117:1697–1701 (2009)]. On page 1698 under “Statistical analysis” and in Table 2, “person-months” should be “person-years.” The authors apologize for these errors.

